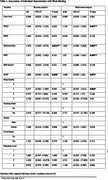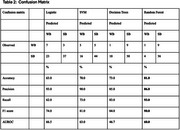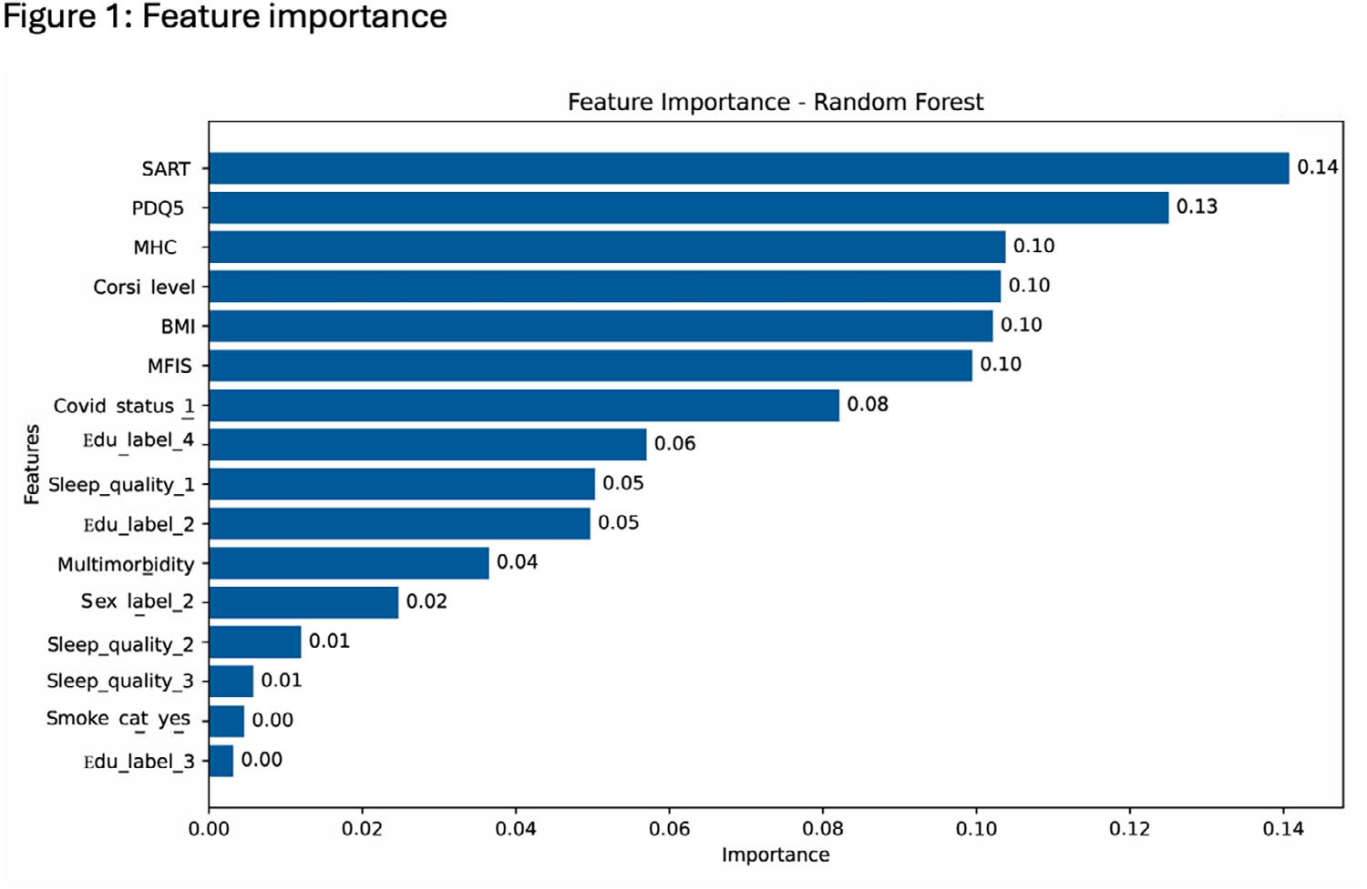# A comparative analysis of analytic approaches for predicting performance on a putative behavioural marker for Alzheimer's disease

**DOI:** 10.1002/alz70857_106450

**Published:** 2025-12-25

**Authors:** Joe Butler, Dennis Boateng, Mario A A Parra, Tamlyn J Watermeyer, Samuel O. Danso, Binding in Neuropsychiatric Disorders (B.I.N.D) Group

**Affiliations:** ^1^ School of Psychology, University of Sunderland, Sunderland, United Kingdom; ^2^ National Institute of Health Applied Research Collaboration North East & Cumbria, Newcastle‐Upon‐Tyne, England, United Kingdom; ^3^ Global Statistical Consult, Accra, Greater Accra Region, Ghana; ^4^ University of Strathcylde, Glasgow, Glasgow, United Kingdom; ^5^ National Institute of Health Applied Research Collaboration North East & Cumbria, Newcastle‐upon‐Tyne, England, United Kingdom; ^6^ Edinburgh Dementia Prevention, Edinburgh, United Kingdom; ^7^ University of Northumbria, Newcastle upon tyne, England, United Kingdom; ^8^ School of Computer Science and Engineering, University of Sunderland, Sunderland, England, United Kingdom; ^9^ University of Edinburgh, Edinburgh, United Kingdom

## Abstract

**Background:**

Remote neurocognitive assessment tools, such as the Visual Short‐Term Memory Binding Task (VSTMBT), offer promising opportunities for assessing Alzheimer's Disease (AD) risk and identifying new population‐level risk factors. State‐of‐the‐art machine learning methods can enhance these tools, providing insights into mechanisms driving risk. This study evaluates three models to examine the impact of self‐reported variables on VSTMBT performance

**Method:**

We categorised participants as strong‐binders (SB – indicative of no pathology; 85.9% of sample) or weak‐binders (WB – indicative of pathology; 14.1%) based on binding‐cost (Parra et al., 2024). Bivariate and multivariate regression analyses were conducted to identify variables with 5% significance and 95% confidence intervals. Continuous variables were summarised using means and standard deviations, while categorical variables were presented as proportions with *p*‐values. Machine Learning models (SVM, RF, DT) were also developed as an alternative analytical approach for validation. Models were trained with an 80/20% split between training and testing.

**Result:**

Both groups shared similar demographics (mean BMI = 24.1kg/m^2^; WB mean age = 28.1, range = 24.8 ‐ 31; SB mean age = 28.6, range = 27.3 ‐ 30.0). Regression models showed significant positive associations of PDQ5 score with higher odds of WB effect (AOR = 1.143; 95% CI: 1.012 – 1.291; *p* = 0.031). Significant positive associations with higher odds of WB effect were observed for multi‐morbidity (AOR = 1.863; 95% CI: 1.082 – 3.208; *p* = 0.025); MHC (AOR = 1.03; 95% CI: 1.082 – 3.208; *p* = 0.049) and SART (AOR = 1.863; 95% CI: 1.000 – 1.020; *p* = 0.075). Consistent sleep quality was marginally associated with lower odds of WB. There were slightly significant lower odds (AOR=0.50, 95% CI: 0.247 ‐ 1.011; *p* = 0.054) See Table 1. Further attribute importance analysis revealed overlapping risk factors identified by our regression models to have high significant odds ratio also being ranked among the top five attributes by the best RF model (Accuracy=81.0, Precision = 86.0; Recall = 93.0; F1 score=90.0; AUROC = 60.0) in Table 3.

**Conclusion:**

Our analysis highlights the value of integrating machine learning with regression approaches to identify VSTMBT predictors and their role in risk stratification. Machine learning complements regression in population‐based AD assessments. Future research should apply these models for individual prediction and early detection.